# Shift work and medically certified sickness absence due to common mental disorders among nursing staff: a prospective cohort study

**DOI:** 10.1093/eurpub/ckag163

**Published:** 2026-09-15

**Authors:** Tove Nilsson, Emma Brulin, Mikko Härmä, Per Gustavsson, Carolina Bigert, Abid Lashari, Jenny Selander, Seth Addo

**Affiliations:** Unit of Occupational Medicine, Institute of Environmental Medicine, Karolinska Institutet, Stockholm, Sweden; Centre for Occupational and Environmental Medicine, Region Stockholm, Stockholm, Sweden; Unit of Occupational Medicine, Institute of Environmental Medicine, Karolinska Institutet, Stockholm, Sweden; Centre for Occupational and Environmental Medicine, Region Stockholm, Stockholm, Sweden; Work ability and Work careers, Finnish Institute of Occupational Health, Helsinki, Finland; Unit of Occupational Medicine, Institute of Environmental Medicine, Karolinska Institutet, Stockholm, Sweden; Centre for Occupational and Environmental Medicine, Region Stockholm, Stockholm, Sweden; Unit of Occupational Medicine, Institute of Environmental Medicine, Karolinska Institutet, Stockholm, Sweden; Centre for Occupational and Environmental Medicine, Region Stockholm, Stockholm, Sweden; Unit of Occupational Medicine, Institute of Environmental Medicine, Karolinska Institutet, Stockholm, Sweden; Unit of Occupational Medicine, Institute of Environmental Medicine, Karolinska Institutet, Stockholm, Sweden; Unit of Occupational Medicine, Institute of Environmental Medicine, Karolinska Institutet, Stockholm, Sweden

## Abstract

Sickness absence due to common mental disorders is rising among healthcare staff, yet evidence on the impact of shift work remains inconsistent. This study examined the association between shift work and the risk of first-time medically certified sickness absence (>14 days) due to common mental disorders (CMDs) among nursing staff. This 11-year prospective cohort study included 25 120 nurses and nursing assistants employed for one year or more between 2008 and 2019 in Region Stockholm. Shift work data were obtained from an employee register, and CMD-related sickness absence (*N* = 2792) from the Swedish Social Insurance Agency, for the study period 2009–2020. Discrete-time proportional hazards models estimated hazard ratios, adjusted for demographic factors and profession. Shift work was assessed in the year preceding sickness absence. Lower hazard ratios (HRs) for sickness absence due to CMDs were found among shift workers with night shifts (HR 0.80, 95% CI 0.72–0.89) and among permanent night workers (HR 0.81, 95% CI 0.65–0.99), compared with day workers. Frequent night shifts (>121/year) were associated with a lower risk (HR 0.77, 95% CI 0.65–0.90) compared with non-night workers. Among non-night workers, frequent quick returns (>49/year) from afternoon shifts (defined as less than 11 hours between shifts) were associated with a lower HR (HR 0.72, 95% CI 0.56–0.92) compared with never having quick returns. Shift work among nursing staff was associated with a lower risk of sickness absence due to common mental disorders. This finding may reflect a selection of healthy individuals for night-shift work.

## Introduction

Sickness absence has increased in Europe since the mid-2010s [1], affecting about 2.9% (5.2 million people) of workers annually [1]. Similarly, in Sweden, sickness absence affects 3.2% of women and 2% of men annually, costing around 40 billion SEK [2]. In Sweden, sickness absence refers to work incapacity due to illness. Absences exceeding 14 calendar days require a medical certificate and are managed by the Swedish Social Insurance Agency, while shorter periods are handled by the employer. Beyond economic and productivity losses, sickness absence entails substantial personal suffering, underscoring the urgent need to identify the factors contributing to it.

Extensive research indicates that psychosocial work factors contribute to stress, burnout and sickness absence [3]. Although inconclusive, previous studies indicate an association between shift work and sickness absence among healthcare workers [4, 5]. In healthcare, where it is necessary to provide medical care and support to patients on a continuous basis, shift work, commonly defined as working hours outside the regular office hours, including early mornings, late evenings, and nights, is unavoidable. Four out of 10 health-care employees in the European Union (EU) and about 44% in Sweden work shifts [6, 7].

A systematic review reveals that estimates of the association between shift work and sickness absence ranged from positive to negative [4]. One reason for this is that previous epidemiological studies lack detailed schedule characteristics and uniform operationalization of sickness absence outcomes [4]. Current research indicates that consecutive night shifts and quick returns (<11 hours between shifts) are associated with a higher risk of nonspecific sickness absence [8, 9]. Conversely, other studies of nurses found no association between night work and sickness absence [10]. Yet another study found that shift work involving nights was associated with fewer long-term sickness absences (>9 days) among hospital personnel compared with day workers [11].

Another reason for variation across studies may be that many previous studies have investigated sickness absence without information on a medically certified diagnosis [8, 9]. Meanwhile, common mental disorders (CMDs) such as anxiety, depression and stress-related disorders are one of the leading causes of sickness absence in Europe [12] and the most common causes in Sweden, especially in the healthcare sector [13]. Research shows that shift work may be associated with an increased risk of CMDs [14–18], however, the findings remain inconsistent.

As CMDs are a leading cause of sickness absence, this study specifically focuses on sickness absence due to CMDs. Using objective, register-based data on shift work and diagnosis-specific sickness absence data for a cohort of about 25 000 nursing staff, we further investigate this complex relationship.

The aim is to investigate the risk of first-time medically certified sickness absence (>14 days) due to CMDs among nursing staff by shift work type.

## Methods

### Study population and analytical sample

**Figure 1:**
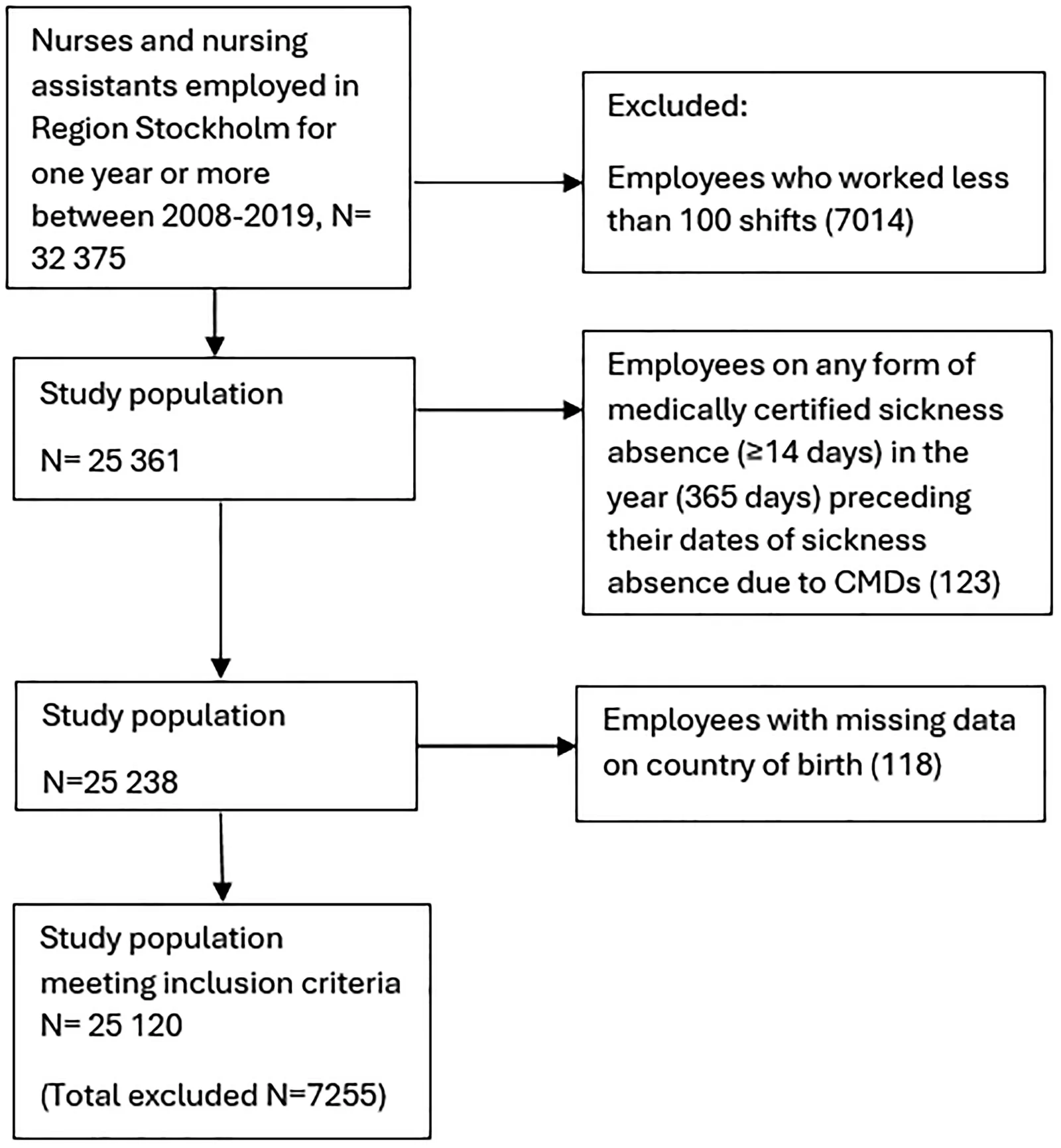
Flowchart for the inclusion and exclusion procedure, cohort for medically certified sickness absence due to common mental disorders (CMDs).

The study population consisted of health-care employees identified from a computerized administrative employee register (HEROMA) in Region Stockholm, who had been employed for one year or more at any time between 1 January 2008 and 31 December 2019. Region Stockholm provides public healthcare, including hospitals, local medical centers, family doctors and maternity clinics. This study was restricted to hospitals and to nursing staff, who are often involved in night and shift work, including nurses, specialist nurses (including midwives), nursing assistants, personal assistants, caregivers and accommodation assistants (grouped under nursing assistants). A total of 6 hospitals offering emergency care were included, including an ophthalmology and a university hospital. Physicians were excluded due to insufficiently detailed data on shift work. We excluded employees who worked fewer than 100 shifts in the preceding year (see below for a description of the analytical model), as this could indicate a medical condition that hindered their ability to work full-time. To reduce the risk of including participants who had a CMD resulting from other health problems, we excluded those who were on medically certified sickness absence (≥14 days) in the year prior to the date of the medically certified sickness absence due to CMDs. We assumed that recent prolonged sickness absence likely reflects existing or ongoing health problems (physical or mental), and a subsequent CMD-related sickness absence may therefore not represent an incident case but rather a CMD secondary to another condition or a continuation or relapse of prior ill health. From the total cohort of nursing staff, we completed an analytical sample of 25 120 employees, 22 455 women and 2665 men (Fig. 1).

The data collection was part of a larger project examining the effects of shift work on various health outcomes [19–24].

### Assessment of exposure

Detailed individual information on the exact start and end times for each shift for the full cohort was obtained from the HEROMA register. The method for aggregating and classifying working time patterns based on register-based working hours for healthcare staff was previously developed in Finland [25] and utilized specifically for HEROMA in our studies investigating shift and night work and different adverse health outcomes [19–24]. All shifts were classified as follows: day shifts (starts after 06:00 and ends no later than 18:00); afternoon shifts (starts after 12:00 and ends later than 18:00, but not a night shift); and night shifts (≥3 hours of work within 22:00–06:00). Furthermore, based on this classification, the cohort participants were for every year, classified as (i) permanent daytime workers, (ii) persons working daytime and/or afternoon shifts but not night shifts, (iii) persons working various shift types including night shifts, and (iv) persons working permanent night shifts. The exposure classification was assessed annually to account for variations over time. An exposure window of one year preceding the outcome was chosen to ensure that individuals had been exposed to shift work for a substantial period, allowing for the development of CMD-related sickness absence.

We calculated annual frequencies of night shifts and quick returns from after night shifts, defined as <28 hours between the end of a night shift and the beginning of the following shift (the following shift could be a day, afternoon or night shift). We also assessed the frequency of ≥3 consecutive night shifts per year. We calculated the frequency of quick returns from afternoon shifts (among non-night workers), defined as <11 hours between the end of an afternoon shift and the beginning of the following shift.

### Data sources and assessment of the outcome

Data on the first spell of medically certified sickness absence due to CMDs (*N* = 2792) was obtained from the Swedish Social Insurance Agency during the study period from January 2009 to December 2020. Long-term sickness absence was defined as sickness absence exceeding 14 consecutive calendar days. ICD-10 diagnosis codes for depression (F32-F33), anxiety (F41) and stress-related disorders (F43) were included.

### Covariates

The model was adjusted for the time-dependent variable age and the fixed variables of profession, sex, and country of birth. Information on age, sex, and country of birth was obtained from Statistics Sweden’s register of the total population. Information on occupation was obtained from the HEROMA register.

### Statistical data analysis

Since the cohort was open and exposure varied over time (individuals may change the type of shift over the study period and thus belong to different exposure groups), descriptive analyses were performed for all included individuals covering a one-year exposure period in 2013, which is approximately the mid-time of the study period.

Hazard ratios (HRs) were estimated by a discrete-time proportional hazards model, subdividing the study period 2009–2020 into 1-year blocks [26], with person-years as the observational unit. The discrete-time proportional hazards model was used because, as noted by Richardson [26], it can simplify longitudinal analysis, especially when time-dependent covariates are involved in cases where tied events occur, and sometimes, even in cases where the exact times of occurrence are known. This approach allows us to estimate the HR for CMD-related sickness absence while flexibly incorporating time-dependent shift-work exposure in the preceding year. The shift pattern was modeled as a time-varying variable. All models were adjusted for year of follow-up, which is inherent in the analytical model, as well as covariates. Models for quick returns were additionally adjusted for the number of night and afternoon shifts in the exposure year, respectively, to limit the confounding effect of the overall frequency of these shifts on the associations between quick returns and sickness absence due to CMDs.

Employees were considered at risk from 1 January of the year following the year when the entry criterion of one year of employment was fulfilled, until their first CMD-related sickness absence, death, or the end of the study period. Thus, non-cases were censored at death or at the end of the study period. Since any participant on any medically certified sickness absence within the last 365 days (1 year) was removed, no participant realistically had a non-CMD-related sickness absence before a CMD-related sickness absence in the same follow-up year. For participants with no CMD-related sickness absence, non-CMD-related sickness absences were treated as non-cases.

The cut-off points for distribution of exposure were based on 1st–25th, 25th–50th, 50th–75th, and >75th percentiles in the distribution of the exposed group. To test the linear trend in the association between exposure and the outcome, we used exposure on a continuous scale in a regression model. The trend tests assessed the risk increases per ten additional times of the different aspects of shift work, for those with incident of CMD-related sickness absence.

Subanalyses were conducted by CMD diagnosis: one for depression (F32–F33) and anxiety (F41) and one for stress-related disorders (F43), due to differing pathophysiological mechanisms. First spells from the general register on sickness absence were used, as in the main analyses. For instance, to assess sickness absence due to depression and anxiety, employees who had their first depression or anxiety spell were extracted and included; subsequent spells or other CMDs were treated as non-incidents. The same exclusion criteria in the main analyses were used, such that employees were excluded if they had another type of CMD in the year prior to diagnosis.

Sensitivity analyses were conducted in two steps: first, by stratifying profession (nurses vs nursing assistants) and second, by excluding day workers and using shift workers without night work as the reference group. These analyses assessed whether results differed between groups and compared with the main analyses. Additional analyses were carried out to test associations with the number of days of sickness absence. A generalized linear model was performed, presenting a linear estimate of the length of sickness absence in relation to different shift types. Finally, a quantitative bias analysis was conducted to address unmeasured confounding for the results of night shift compared with day work only. This was done by specifying and testing healthy worker selection and family living arrangements (whether living with children or otherwise) as unmeasured confounders. We then ran a grid-based quantitative bias analysis to capture different possible HR values, varying biases, and plausible scenarios for both confounders. We used SAS software version 9.4 for Windows (SAS Institute Inc, Cary, NC, USA), with statistical significance set at *P* < .05 for all statistical analyses.

### Ethics approval

Ethical approval for the study was granted by the Ethical Review Board in Stockholm and the Swedish Ethical Review Authority (2016/2490–31; 2017/1157–32, 2022-06828-02).

### Patient consent statement

Informed consent for participation in the study was not required for register-based research.

## Results

The characteristics of the study population for the year 2013 (approximately mid-time of the study period) are presented in Table 1. Characteristics are further subdivided by shift work types.

**Table 1:** Characteristics of the population for the year 2013 (*N* 12 400), subdivided by work schedule based on how each person worked during that year. In all analyses, cohort subjects were classified dynamically for each year of follow-up.

	Full cohort		**Day work only** ^a^		**Shift work, without night shift** ^b^		**Shift work, with night shift** ^c^		**Night work only** ^d^	
	*N*	(%)	*N*	%	*N*	%	*N*	%	*N*	%
**Total**	12 400	100	2982	24.0	5140	41.5	3557	28.7	721	5.8
**Sex**										
Women	11 285	91.0	2792	93.6	4722	91.9	3110	87.4	661	91.7
Men	1115	9.0	190	6.4	418	8.1	447	12.6	60	8.3
**Age**										
≤40 years	4738	38.2	498	16.7	2226	43.3	1836	51.6	178	24.7
41–50 years	3255	26.3	856	28.7	1294	25.2	908	25.5	197	27.3
>50 years	4407	35.5	1628	54.6	1620	31.5	813	22.9	346	48.0
**Country of birth**										
Sweden	9949	80.2	2533	84.9	3992	77.7	2875	80.8	549	76.1
Nordic countries (except Sweden)	669	5.4	191	6.4	239	4.6	181	5.1	58	8.0
Europe (except Nordic countries)	561	4.5	94	3.2	255	5.0	175	4.9	37	5.1
Outside Europe	1221	9.9	164	5.5	654	12.7	326	9.2	77	10.7
**Profession**										
Nursing assistants^e^	4722	38.1	834	28.0	2459	47.8	1076	30.3	353	49.0
Nurses, including midwives	7678	61.9	2148	72.0	2681	52.2	2481	69.7	368	51.0

a Day work: starts after 06:00 and ends no later than 18:00.

b At least one afternoon shift (starts after 12:00 and ends later than 18:00, but not a night shift).

c At least one night shift (at least 3 hours within 22:00 hours–06:00 hours), but not only night work.

d Only night work (no day work or afternoon shifts).

e Nursing assistants and caregivers.

Among 12 400 employees, 2982 had permanent day work, 5140 worked shifts without nights, 3557 had shifts including nights and 721 worked only nights. The majority were women (91%) and born in Sweden (80%). The cohort comprised 62% nurses and 38% nursing assistants. The ages of participants, after inclusion and exclusion, ranged from 19 years to 74 years. Older employees (50+ years) were more common in the day work-only (54.6%) and night-work-only groups (48%), while workers with various shift types tended to be younger. Nurses were more prevalent than nursing assistants across all shift types, especially in the day-only group.

The 25 120 participants contributed to 136 880 person-years from January 2009 to December 2020. During the study period, 2792 cases of medically certified sickness absence occurred, with a mean duration of 165 days for first spells. Diagnoses included 106 cases of major depression, 554 of depression, 1746 stress-related disorders and 386 of anxiety.

### Effect of shift work on first-time medically certified sickness absence due to CMDs

The results of the analysis examining the association between shift work characteristics and first-time medically certified CMD-related sickness absence are presented in Table 2.

**Table 2:** Hazard ratio (HR) for first-time medically certified sickness absence due to common mental disorders (*N* = 2792, ICD: F32-33, F41 and F43) in association with various aspects of shift work (*N* = 25 120) the preceding year among healthcare employees during January 2009–December 2020

Exposure	Person years	No. of cases	Crude HR	Adjusted HR^a^ (95% CI)
**Type of shift work (the full cohort)**				
Day work only	34 637	684	1	1
Shift work, without night shift	57 286	1299	1.15 (1.05–1.26)	0.94 (0.85–1.04)
Shift work, with night shift	37 725	703	0.93 (0.83–1.03)	0.80 (0.72–0.89)
Night work only	7232	106	0.73 (0.59–0.90)	0.81 (0.65–0.99)
**Frequency of night shifts (the full sample)**				
Never night shifts^b^	91 923	1983	1	1
1–18	11 760	226	0.88 (0.76–1.00)	0.83 (0.72–0.96)
19–49	11 570	214	0.84 (0.73–0.96)	0.83 (0.71–0.95)
50–121	11 205	201	0.82 (0.71–0.95)	0.83 (0.72–0.96)
>121	10 422	168	0.73 (0.62–0.85)	0.77 (0.65–0.90)
Trend test				
*P*-value trend			.102	.449
**Frequency of ≥3 consecutive night shifts (includes only those with night shifts)** ^c^				
0 times	10 308	192	1	1
1–4	9968	198	1.06 (0.87–1.29)	1.05 (0.86–1.28)
5–12	8333	145	0.92 (0.74–1.14)	0.94 (0.76–1.17)
13–21	8560	129	0.81 (0.64–1.00)	0.83 (0.66–1.04)
>21	7788	145	0.98 (0.79–1.22)	1.04 (0.84–1.30)
Trend test				
*P*-value trend			.511	.765
**Quick returns (<28 hours) from night shifts (includes only those with night shifts)** ^c^				
No quick returns from night shifts	1404	37	1	1
1–9	10 443	202	0.73 (0.52–1.05)	0.74 (0.53–1.07)
10–28	11 509	205	0.66 (0.47–0.95)	0.69 (0.48–1.01)
29–69	11 122	182	0.61 (0.44–0.89)	0.66 (0.40–1.11)
>69	10 479	183	0.65 (0.46–0.94)	0.72 (0.36–1.42)
Trend test				
*P*-value trend			.296	.917
**Quick returns (<11 hours) from afternoon shifts (includes only non-night workers)** ^b^				
No quick returns from afternoon shifts	7491	176	1	1
1–17	10 810	231	0.89 (0.73–1.09)	0.85 (0.69–1.06)
18–32	10 427	258	1.03 (0.85–1.25)	0.93 (0.75–1.16)
33–49	12 608	316	1.05 (0.88–1.27)	0.94 (0.75–1.17)
>49	15 964	318	0.84 (0.70–1.01)	0.72 (0.56–0.92)
Trend test				
*P*-value trend			.246	.114

a Adjusted for the time-dependent variables age and the fixed variables profession, sex, and country of birth. Quick return analyses were adjusted for night and afternoon shift counts, respectively. Adjustment for calendar year of follow-up is inherent in the analytical model.

b Those who worked day and/or afternoon shifts, but no night shifts (non-night workers).

c Delimited to those who worked nights (shift work with night shifts or permanent night work).

The results in the following text are presented as adjusted hazard ratios (HRs). A lower HR of CMD-related sickness absence was found among those who, during the preceding year, had shift work with nights (HR 0.80, 95% CI 0.72–0.89), and worked only nights (HR 0.81, 95% CI 0.65–0.99), compared with day workers.

The analysis of the frequency of night shifts showed that all groups were associated with a decrease in HR of CMD-related sickness absence. The HR was lowest among those working more than 121 night shifts (the highest quartile) in the preceding year (HR 0.77, 95% CI 0.65–0.90) compared with those who did not work nights. Among those who had night shifts, we found no associations between a high frequency of working three or more consecutive night shifts and quick returns from night shifts and sickness absence. Among non-night workers (i.e. day workers and shift workers without night shifts), frequently (>49 times in 1 year) had quick returns from afternoon shifts (<11 hours) was associated with a decreased HR of sickness absence (HR 0.72, 95% CI 0.56–0.92) compared with those who had no quick returns.

Sub-analyses were carried out for the diagnoses of depression and anxiety, and for stress-related disorders (Tables S1 and S2). The sub-analyses showed a pattern resembling the combined analysis, with some variations. For depression and anxiety, there was a decreased HR of sickness absence among those who only worked night shifts (HR 0.65, 95% CI 0.47–0.88) compared to day workers only, while for stress-related disorders, those working shifts with nights had a decreased HR (HR 0.82, 95% CI 0.72–0.93).

Sensitivity analyses for nurses and nursing assistants aligned with the main results (Tables S3 and S4). Night work only was associated with a decreased HR of CMD-related sickness absence compared to day work for both nurses (HR 0.73, 95% CI 0.54–0.97) and nursing assistants (HR 0.68, 95% CI 0.51–0.91). More frequent night shifts (quartiles) were associated with lower HR of sickness absence than for those who never worked night shifts the preceding year in both groups, consistent with the main analyses. Further excluding day only workers, night shift work was still associated with decreased HRs of CMD-related sickness absence for nurses (HR 0.69, 95% CI 0.51–0.91) and nursing assistants (HR 0.69, 95% CI 0.52–0.89) compared with shift work without nights and the results were consistent across both groups and with the main analyses (Tables S5 and S6).

Additional analyses using linear estimation showed that the mean length of CMD-related sickness absence (the number of days) was lower among those who worked shifts without nights (33.15 days less, *P* = .01) and shifts with nights (28.75 days less, *P* = .05), compared with day workers only. The mean length of sickness absence for night workers was not different compared with that of day workers (*P* = .17). Similarly, analyses of quick returns from night and afternoon shifts, and ≥3 consecutive night shifts did not differ between the groups (see Table S7).

Lastly, we ran a quantitative bias analysis for unmeasured confounding, specifying healthy worker selection and family living arrangements, for night work and day work only. We assumed, logically, that healthier workers are selected more for night work compared to day work, and that they are likely to be associated with decreased sickness absence due to CMDs. For family living arrangements, we assumed that workers with children are likely to have more day work than night work compared with those who have no children. Family demands on married workers with children are more likely to be associated with increased sickness absence due to CMDs.

We suggested possible adjusted HR value ranges for healthy worker selection as: night workers (0.5–0.8), day workers (0.3–0.6), and risk of sickness absence due to CMDs (0.6–0.9). Those for family living arrangements were night workers (0.3–0.6), day workers (0.4–0.7), and risk of sickness absence due to CMDs (1.1–1.5). The results showed combined bias-adjusted HRs for both confounders of 0.79 (5^th^ percentile), 0.87 (50^th^ percentile), and 1.01 (95^th^ percentile). This means that taking possible ranges of unmeasured confounding for both covariates into consideration, most of the distribution remained below 1.0, ranging from 0.79 to 1.01 with a median of 0.87. This also means that under most reasonable assumptions about bias, night shift was still associated with decreased sickness absence due to CMDs compared with day work.

## Discussion

This study was initiated to investigate the complex relationship between shift work and medically certified sickness absence exceeding 14 days due to CMDs. We found that nursing staff working rotating shifts including nights or only night shifts had a lower HR for sickness absence compared with day workers. Higher night shift intensity and frequent quick returns from afternoon shifts were also associated with reduced HR. These results both support [4, 11] and contradict [8, 10] previous studies.

The inconsistencies across studies may stem from several methodological and contextual factors. First, the operationalization of sickness absence differs across countries and studies, which likely impacts the results [11]. For instance, several studies are conducted in the Nordic countries and while these nations share many similarities, differences remain in areas such as social insurance, sickness benefits and working time norms [27–29]. Sweden’s 40-hour workweek exceeds Denmark’s, Norway’s and Finland’s norm of about 37. Secondly, the duration of sickness absence may vary across studies, potentially affecting the outcome. For instance, one Norwegian study shows that the associations between shift work and sickness absence differ when the outcome is short-term (i.e. 1–8 days) compared with long-term (>9 days) [11]. Whether the employee could call in ill or needed a medical certificate stating they were ill could also be important for the results, as the latter may imply a greater effort and require a clinically relevant reduction in work ability. Our study includes sickness absence cases exceeding 14 days that are medically certified by a physician, which we interpret as reflecting a greater impairment of work ability. Furthermore, while the majority of studies use non-diagnose-specific sickness absence [8, 9], we only include those due to CMD. Third, follow-up durations vary across studies. For instance, while one study [10] used a 1-month follow-up, another study [8], like ours, assessed the risk of sickness absence after one year of exposure. Prior research with longer follow-up suggests that mental health impacts from shift work may emerge after several years [18, 30]. Overall, methodological and cross-country differences complicate comparisons of studies assessing shift work and sickness absence; this study therefore makes an important contribution.

One probable explanation for our results is a selection effect rather than the idea that night shifts protect against sickness absence. Our results may thus reflect a healthy worker effect, suggesting that individuals with mental health problems may change out of night and shift work, either by their own choice or because of medical screening of night workers, as suggested in previous studies [11]. This hypothesis is also supported by a longitudinal study linking declining mental health to leaving night work [31] and by a study showing that employees with health problems reduced night shifts [32]. Future research should pay particular attention to testing the healthy worker effect. This could, for instance, be done by moving away from observational studies which have been used in most previous studies [4, 15] toward more quasi-experimental designs to determine whether changes in shift work schedules affect the risk of sickness absence due to CMDs.

Swedish employers are required to offer medical check-ups for night workers [33], and the development of sleep or mood symptoms may necessitate advising against continued night work. These medical check-ups may thus act as an eye-opener and support workers in transitioning away from night shifts. The role of medical check-ups for preventing adverse effects of night work should be further investigated. Additionally, further research is needed to better understand the underlying mechanisms and causal pathways.

Another probable explanation for why shift work is associated with reduced CMD-related absence is a lighter workload, as nurses’ strain contributes to poor health, CMDs, and sickness absence [34]. Day shifts involve more patient and staff interactions, heavier administrative tasks, and a faster workflow, leading to a higher workload than at night [35]. A study found that night shifts were less demanding in terms of workload and emotional strain than day shifts [36]. Furthermore, in Sweden, national and regional collective agreements in the healthcare sector often provide reduced weekly working hours for employees working night shifts, which may enhance opportunities for recovery and, in turn, contribute to lower CMD-related absence [37].

### Strengths and limitations

A strength of this study is the use of objective register-based data to examine shift work and its associations with sickness absence. An additional strength is that the analyses include stratifications and sensitivity analyses to assess the study’s validity. Furthermore, we focused on nurses and nursing assistants in Region Stockholm to ensure a well-defined study population with similar educational and occupational qualifications. We included only individuals with one year or more of employment to increase the likelihood of sufficient exposure to shift work and excluded those with prior sickness absence due to other diagnoses to reduce the influence of preexisting conditions.

This study also has several limitations. First, the inability to adjust for several potentially important confounders, including physical health status, health-related behaviors, and family circumstances, hampers the study’s generalizability. Moreover, work-related factors such as the ability to influence work schedules are likely to be important in this context. Previous research suggests that greater control over working hours may attenuate the adverse effects of shift work on sickness absenteeism outcomes [38]. However, due to the register-based design, information on these covariates was limited and could not be included in the analysis. Meanwhile, as the data covered a defined health-care region over a long period, it is likely that a majority of workers have experienced aspects of self-roasting.

Second, sickness absence diagnoses and duration vary by national social insurance policies. For instance, when ICD code 43.8 (stress-related exhaustion disorder) was introduced in Sweden in 2010, depression-related absences declined while exhaustion-related absences increased [39]. We addressed this by analyzing specific diagnoses and sickness absence days, and adjusting for calendar time, to enhance study validity.

Third, non-CMD-related sickness absence during follow-up was not treated as a censoring event, which we acknowledge as a limitation, as individuals on sickness absence for other reasons are not at risk of CMD-related sickness absence during that period.

Fourth, in this study, we applied a 1-year follow-up. Following previous literature [8, 10, 15], we concluded that a one-year exposure window represents a reasonable compromise for capturing the potential effects of shift work, balancing the possibility of more acute effects with the need to reflect longer-term influences. Furthermore, longer follow-up periods are methodologically challenging, as individuals may change shift types over time [32, 40]. Based on this, we assessed exposure to shift work during the year prior to the first registered sickness absence.

## Conclusion

Shift work among nursing staff was associated with lower hazard ratios for sickness absence due to CMDs compared with day workers. These findings may reflect a selection effect, suggesting that individuals with mental health problems may leave shift work, either voluntarily or through the employer’s mandatory medical check-ups. Another reason may be a lower workload during day shifts than during night shifts. Taken together, the results indicate the need for public health and workplace policies to account for selection effects when evaluating mental health risks and designing preventive strategies for shift-working populations.

Key points

- This study investigated whether shift work is associated with the risk of first-time medically certified sickness absence (>14 days) due to common mental disorders (CMDs) among nursing staff.
- Nursing staff working night-shifts had a lower risk of sickness absence due to CMDs than those working daytime-only, and—contrary to previous research—more frequent quick returns from afternoon shifts were also linked to a lower risk of CMD-related sickness absence.
- These findings may reflect a healthy worker effect, suggesting that individuals with health issues may be selected out of night and shift work, either by their own choice or because of the employer’s mandatory medical check-ups of night-shift workers.
- The results highlight the need for public health and workplace policies to account for selection effects when evaluating mental health risks and designing preventive strategies for shift-working populations.

## Supplementary Material

ckag163_Supplementary_Data

## Data Availability

The sensitive and confidential data used in this study cannot be made public under the General Data Protection Regulation, Swedish law SFS 2018:218, the Swedish Data Protection Act, the Swedish Ethical Review Act and the Public Access to Information and Secrecy Act.
