# Supplementary material for: Shift work and medically certified sickness absence due to common mental disorders among nursing staff: a prospective cohort study
Source: Eur J Public Health. 2026 Sep 15;36(5):ckag163. doi: 10.1093/eurpub/ckag163 (PMC13576068; doi:10.1093/eurpub/ckag163)
Supplement: Supplementary file 1 [file ckag163_supplementary_data.docx]

**Supplementary material**

**Table S1.** Discrete-time proportional hazards model with estimated hazard ratio (HR) for first-time medically certified sickness absence due to **depression and anxiety** (N=1355, ICD: F32-33 and F41) in association with various aspects of shift work among health care employees during the study period January 2009 - December 2020. We analysed the outcome as functions of working hour characteristics in the preceding year. The cut-off points for the distribution of exposure were based on quartiles in the distribution of the exposed group.

| Exposure | Person years | No. of cases | Crude HR | Adjusted HR^a^ (95% CI) |
| --- | --- | --- | --- | --- |
| **Type of shift work** |  |  |  |  |
| Day work only | 39 113 | 312 | 1 | 1 |
| Shift work, without night shifts | 63 940 | 659 | 1.29(1.13-1.48) | 0.93(0.80-1.07) |
| Shift work, with night shifts | 41 041 | 336 | 1.02(0.87-1.19) | 0.88(0.75-1.03) |
| Night work only | 8056 | 48 | 0.74(0.54-0.99) | 0.65(0.47-0.88) |
| **Frequency of night shifts** |  |  |  |  |
| Never night shifts^b^ | 103 053 | 971 | 1 | 1 |
| 1-18 | 12 755 | 107 | 0.88(0.72-1.07) | 0.81(0.66-0.98) |
| 19-49 | 12 462 | 104 | 0.88(0.71-1.07) | 0.84(0.68-1.03) |
| 50-121 | 12 367 | 95 | 0.81(0.65-0.99) | 0.82(0.66-1.00) |
| >121 | 11 513 | 78 | 0.72(0.56-0.90) | 0.75(0.59-0.94) |
| Trend test |  |  |  |  |
| p-value trend |  |  | 0.086 | 0.470 |
| **Frequency of ≥3 consecutive night shifts^c^** |  |  |  |  |
| 0 times | 11 320 | 89 | 1 | 1 |
| 1-4 | 10 743 | 100 | 1.19(0.89-1.58) | 1.12(0.84-1.50) |
| 5-12 | 9028 | 71 | 1.01(0.74-1.37) | 0.98(0.72-1.34) |
| 13-21 | 9407 | 66 | 0.90(0.65-1.23) | 0.93(0.67-1.28) |
| >21 | 8599 | 58 | 0.87(0.62-1.20) | 0.92(0.65-1.28) |
| Trend test |  |  |  |  |
| p-value trend |  |  | 0.089 | 0.390 |
| **Quick returns (<28 hours) from night shifts^c^** |  |  |  |  |
| No quick returns from night shifts | 1537 | 15 | 1 | 1 |
| 1-9 | 11 327 | 100 | 0.90(0.54-1.61) | 0.90(0.54-1.62) |
| 10-28 | 12 409 | 95 | 0.78(0.47-1.40) | 0.83(0.48-1.54) |
| 29-69 | 12 281 | 94 | 0.78(0.47-1.41) | 0.98(0.47-2.13) |
| >69 | 11 543 | 80 | 0.71(0.42-1.28) | 1.01(0.38-2.77) |
| Trend test |  |  |  |  |
| p-value trend |  |  | 0.060 | 0.335 |
| **Quick returns (<11 hours) from afternoon shifts^b^** |  |  |  |  |
| No quick returns from afternoon shifts | 8597 | 98 | 1 | 1 |
| 1-17 | 12 273 | 121 | 0.86(0.66-1.13) | 0.80(0.60-1.08) |
| 18-32 | 11 658 | 125 | 0.94(0.72-1.23) | 0.79(0.59-1.07) |
| 33-49 | 13 977 | 146 | 0.92(0.71-1.19) | 0.74(0.55-1.01) |
| >49 | 17 451 | 169 | 0.84(0.66-1.08) | 0.64(0.47-0.90) |
| Trend test |  |  |  |  |
| p-value trend |  |  | 0.364 | 0.073 |

^a^Adjusted for the time-dependent variables age and the fixed variables profession, sex, and country of birth. Quick return analyses were adjusted for night and afternoon shift counts, respectively. Adjustment for calendar year of follow-up is inherent in the analytical model.
^b^Those who worked day and/or afternoon shifts, but no night shifts.
^c^Delimited to those who work nights (shift work with night shifts or permanent night work)

**Table S2.** Discrete-time proportional hazards model with estimated hazard ratio (HR) for first-time medically certified sickness absence due to **stress-related disorders** (N=2122 ICD: F43) in association with various aspects of shift work among health care employees during the study period January 2009 - December 2020. We analysed the outcome as functions of working hour characteristics in the preceding year. The cut-off points for the distribution of exposure were based on quartiles in the distribution of the exposed group.

| Exposure | Person years | No. of cases | Crude HR | Adjusted HR^a^ (95% CI) |
| --- | --- | --- | --- | --- |
| **Type of shift work** |  |  |  |  |
| Day work only | 37 041 | 541 | 1 | 1 |
| Shift work, without night shift | 61 925 | 984 | 1.09(0.98-1.21) | 1.00(0.90-1.12) |
| Shift work, with night shift | 40 073 | 516 | 0.86(0.76-0.97) | 0.82(0.72-0.93) |
| Night work only | 7713 | 81 | 0.71(0.56-0.89) | 0.94(0.74-1.19) |
| **Frequency of night shifts** | | | | |
| Never night shifts^b^ | 98 966 | 1525 | 1 | 1 |
| 1-18 | 12 467 | 176 | 0.90(0.77-1.05) | 0.91(0.77-1.06) |
| 19-49 | 12 210 | 138 | 0.71(0.59-0.84) | 0.74(0.61-0.87) |
| 50-121 | 11 968 | 157 | 0.84(0.71-0.98) | 0.86(0.72-1.01) |
| >121 | 11 141 | 126 | 0.71(0.59-0.85) | 0.73(0.61-0.88) |
| Trend test |  |  |  |  |
| p-value trend |  |  | 0.325 | 0.271 |
| **Frequency of ≥3 consecutive night shifts^c^** | | | | |
| 0 times | 10 938 | 145 | 1 | 1 |
| 1-4 | 10 531 | 138 | 0.98(0.77-1.23) | 1.02(0.81-1.29) |
| 5-12 | 8887 | 102 | 0.84(0.65-1.09) | 0.90(0.70-1.16) |
| 13-21 | 9115 | 102 | 0.84(0.65-1.08) | 0.84(0.65-1.09) |
| >21 | 8315 | 110 | 0.97(0.75-1.24) | 1.00(0.78-1.29) |
| Trend test |  |  |  |  |
| p-value trend |  |  | 0.814 | 0.695 |
| **Quick returns (<28 hours) from night shifts^c^** | | | | |
| No quick returns from night shifts | 1502 | 125 | 1 | 1 |
| 1-9 | 11 046 | 156 | 0.84(0.56-1.32) | 0.89(0.59-1.39) |
| 10-28 | 12 173 | 139 | 0.67(0.44-1.04) | 0.71(0.46-1.14) |
| 29-69 | 11 883 | 141 | 0.70(0.47-1.10) | 0.71(0.39-1.30) |
| >69 | 11 182 | 136 | 0.71(0.47-1.11) | 0.69(0.32-1.53) |
| Trend test |  |  |  |  |
| p-value trend |  |  | 0.625 | 0.603 |
| **Quick returns (<11 hours) from afternoon shifts^b^** | | | | |
| No quick returns from afternoon shifts | 8232 | 149 | 1 | 1 |
| 1-17 | 11 786 | 191 | 0.86(0.70-1.07) | 0.84(0.67-1.07) |
| 18-32 | 11 327 | 197 | 0.92(0.75-1.14) | 0.87(0.69-1.12) |
| 33-49 | 13 579 | 237 | 0.94(0.76-1.15) | 0.88(0.69-1.14) |
| >49 | 17 016 | 210 | 0.67(0.55-0.83) | 0.61(0.47-0.81) |
| Trend test |  |  |  |  |
| p-value trend |  |  | 0.1780 | 0.0698 |

^a^Adjusted for the time-dependent variables age and the fixed variables profession, sex, and country of birth. Quick return analyses were adjusted for night and afternoon shift counts, respectively. Adjustment for calendar year of follow-up is inherent in the analytical model.
^b^Those who worked day and/or afternoon shifts, but no night shifts.
^c^Delimited to those who work nights (shift work with night shifts or permanent night work)

**Table S3.** Discrete-time proportional hazards model with estimated hazard ratio (HR) for first-time medically certified sickness absence due to CMDs for **nurses only** in association with various aspects of shift work among health care employees during the study period January 2009 - December 2020. We analysed the outcome as functions of working hour characteristics in the preceding year. The cut-off points for the distribution of exposure were based on quartiles in the distribution of the exposed group.

| Exposure | Person Years | No. of cases | Crude HR | Adjusted HR^a^ (95% CI) |
| --- | --- | --- | --- | --- |
| **Type of shift work (the full sample)** | | | | |
| Day work only | 24251 | 447 | 1 | 1 |
| Shift work, without night shift | 27919 | 580 | 1.14(1.00-1.29) | 1.06(0.93-1.21) |
| Shift work, with night shift | 24255 | 405 | 0.89(0.78-1.02) | 0.86(0.74-0.99) |
| Night work only | 3589 | 49 | 0.74(0.54-0.98) | 0.73(0.54-0.97) |
|  |  |  |  |  |
| **Frequency of night shifts (the full sample)** | | | | |
| Never night shifts^b^ | 52170 | 1027 | 1 | 1 |
| 1-18 | 7234 | 123 | 0.85(0.70-1.02) | 0.82(0.68-0.99) |
| 19-49 | 7220 | 123 | 0.85(0.70-1.02) | 0.83(0.68-1.00) |
| 50-121 | 6730 | 111 | 0.82(0.67-1.00) | 0.83(0.68-1.01) |
| >121 | 6660 | 97 | 0.73(0.59-0.89) | 0.76(0.61-0.93) |
| Trend test |  |  |  |  |
| p-value trend |  |  | 0.372 | 0.894 |
| **Frequency of ≥3 consecutive night shifts (includes only those with night shifts)^c^** | | | | |
| 0 times | 6877 | 108 | 1 | 1 |
| 1-4 | 5499 | 94 | 1.08(0.82-1.43) | 1.07(0.81-1.41) |
| 5-12 | 5706 | 93 | 1.02(0.78-1.35) | 1.04(0.79-1.38) |
| 13-21 | 4773 | 74 | 0.98(0.73-1.32) | 1.02(0.76-1.37) |
| >21 | 4989 | 85 | 1.07(0.81-1.42) | 1.16(0.87-1.54) |
| Trend test |  |  |  |  |
| p-value trend |  |  | 0.968 | 0.470 |
| **Quick returns (<28 hours) from night shifts (includes only those with night shifts)^c^** | | | | |
| No quick returns from night shifts | 821 | 21 | 1 | 1 |
| 1-9 | 6851 | 114 | 0.64(0.41-1.05) | 0.63(0.40-1.03) |
| 10-28 | 6942 | 124 | 0.68(0.44-1.12) | 0.57(0.36-0.95) |
| 29-69 | 6610 | 97 | 0.56(0.36-0.93) | 0.33(0.18-0.62) |
| >69 | 6620 | 98 | 0.57(0.36-0.93) | 0.21(0.09-0.51) |
| Trend test |  |  |  |  |
| p-value trend |  |  | 0.415 | 0.020 |
| **Quick returns (<11 hours) from afternoon shifts (includes only non-night workers)^b^** | | | | |
| No quick returns from afternoon shifts | 4548 | 109 | 1 | 1 |
| 1-17 | 1917 | 22 | 0.47(0.29-0.72) | 0.46(0.28-0.73) |
| 18-32 | 3298 | 75 | 0.92(0.69-1.24) | 0.91(0.66-1.26) |
| 33-49 | 4690 | 97 | 0.84(0.64-1.10) | 0.79(0.57-1.09) |
| >49 | 13476 | 277 | 0.84(0.68-1.05) | 0.74(0.53-1.04) |
| Trend test |  |  |  |  |
| p-value trend |  |  | 0.308 | 0.119 |

^a^Adjusted for the time-dependent variables age and the fixed variables profession, sex, and country of birth. Quick return analyses were adjusted for night and afternoon shift counts, respectively. Adjustment for calendar year of follow-up is inherent in the analytical model.
^b^Those who worked day and/or afternoon shifts, but no night shifts.
^c^Delimited to those who work nights (shift work with night shifts or permanent night work)

**Table S4.** Discrete-time proportional hazards model with estimated hazard ratio (HR) for first-time medically certified sickness absence due to CMDs for **nursing assistants only** in association with various aspects of shift work among health care employees during the study period January 2009 - December 2020. We analysed the outcome as functions of working hour characteristics in the preceding year. The cut-off points for the distribution of exposure were based on quartiles in the distribution of the exposed group.

| Exposure | Person Years | No. of cases | Crude HR | Adjusted HR^a^ (95% CI) |
| --- | --- | --- | --- | --- |
| **Type of shift work (the full sample)** | | | | |
| Day work only | 10386 | 237 | 1 | 1 |
| Shift work, without night shift | 29367 | 719 | 1.06(0.92-1.23) | 0.98(0.85-1.15) |
| Shift work, with night shift | 13470 | 298 | 0.94(0.79-1.12) | 0.85(0.71-1.01) |
| Night work only | 3643 | 57 | 0.67(0.50-0.89) | 0.68(0.51-0.91) |
|  |  |  |  |  |
| **Frequency of night shifts (the full sample)** | | | | |
| Never night shifts^b^ | 39753 | 956 | 1 | 1 |
| 1-18 | 4324 | 97 | 0.91(0.73-1.12) | 0.84(0.67-1.03) |
| 19-49 | 4221 | 103 | 0.99(0.80-1.20) | 0.92(0.75-1.13) |
| 50-121 | 4498 | 81 | 0.74(0.59-0.93) | 0.75(0.59-0.93) |
| >121 | 4070 | 74 | 0.74(0.58-0.93) | 0.79(0.62-1.00) |
| Trend test |  |  |  |  |
| p-value trend |  |  | 0.044 | 0.285 |
| **Frequency of ≥3 consecutive night shifts (includes only those with night shifts)^c^** | | | | |
| 0 times | 3431 | 84 | 1 | 1 |
| 1-4 | 3768 | 85 | 0.90(0.67-1.22) | 0.91(0.67-1.23) |
| 5-12 | 3149 | 64 | 0.82(0.59-1.14) | 0.85(0.61-1.18) |
| 13-21 | 3566 | 56 | 0.64(0.46-0.90) | 0.69(0.49-0.97) |
| >21 | 3199 | 66 | 0.82(0.59-1.14) | 0.92(0.66-1.27) |
| Trend test |  |  |  |  |
| p-value trend |  |  | 0.057 | 0.271 |
| **Quick returns (<28 hours) from night shifts (includes only those with night shifts)^c^** | | | | |
| No quick returns from night shifts | 583 | 16 | 1 | 1 |
| 1-9 | 3852 | 90 | 0.84(0.51-1.48) | 0.82(0.49-1.45) |
| 10-28 | 4183 | 95 | 0.80(0.49-1.42) | 0.74(0.42-1.38) |
| 29-69 | 4412 | 77 | 0.64(0.38-1.14) | 0.53(0.21-1.35) |
| >69 | 4083 | 77 | 0.67(0.40-1.19) | 0.53(0.17-1.71) |
| Trend test |  |  |  |  |
| p-value trend |  |  | 0.060 | 0.332 |
| **Quick returns (<11 hours) from afternoon shifts (includes only non-night workers)^b^** | | | | |
| No quick returns from afternoon shifts | 2943 | 67 | 1 | 1 |
| 1-17 | 5719 | 141 | 1.07(0.80-1.44) | 1.03(0.75-1.41) |
| 18-32 | 6151 | 177 | 1.24(0.94-1.66) | 1.17(0.86-1.61) |
| 33-49 | 6764 | 178 | 1.15(0.87-1.53) | 1.08(0.78-1.50) |
| >49 | 7794 | 156 | 0.87(0.66-1.17) | 0.82(0.58-1.17) |
| Trend test |  |  |  |  |
| p-value trend |  |  | 0.168 | 0.147 |

^a^Adjusted for the time-dependent variables age and the fixed variables profession, sex, and country of birth. Quick return analyses were adjusted for night and afternoon shift counts, respectively. Adjustment for calendar year of follow-up is inherent in the analytical model.
^b^Those who worked day and/or afternoon shifts, but no night shifts.
^c^Delimited to those who work nights (shift work with night shifts or permanent night work)

**Table S5.** Discrete-time proportional hazards model with estimated hazard ratio (HR) for first-time medically certified sickness absence due to CMDs for **nurses only** in association with type of shift work and frequency of night shift among health care employees during the study period January 2009 - December 2020. We analysed the outcome as functions of working hour characteristics in the preceding year. The cut-off points for the distribution of exposure were based on quartiles in the distribution of the exposed group.

| Exposure | Person Years | No. of cases | Crude HR | Adjusted HR^a^ (95% CI) |
| --- | --- | --- | --- | --- |
| **Type of shift work (the full sample)** | | | | |
| Shift work, without night shift | 27919 | 580 | 1 | 1 |
| Shift work, with night shift | 24255 | 405 | 0.79(0.69-0.89) | 0.81(0.72-0.92) |
| Night work only | 3589 | 49 | 0.65(0.48-0.86) | 0.69(0.51-0.91) |
|  |  |  |  |  |
| **Frequency of night shifts (the full sample)** | | | | |
| Never night shifts^b^ | 27919 | 580 | 1 | 1 |
| 1-18 | 7234 | 123 | 0.81(0.66-0.97) | 0.80(0.66-0.97) |
| 19-49 | 7220 | 123 | 0.80(0.66-0.97) | 0.82(0.67-0.99) |
| 50-121 | 6730 | 111 | 0.78(0.63-0.95) | 0.82(0.67-1.00) |
| >121 | 6660 | 97 | 0.69(0.55-0.85) | 0.74(0.59-0.92) |
| Trend test |  |  |  |  |
| p-value trend |  |  |  |  |

^a^Adjusted for the time-dependent variables age and the fixed variables profession, sex, and country of birth. Quick return analyses were adjusted for night and afternoon shift counts, respectively. Adjustment for calendar year of follow-up is inherent in the analytical model.

^b^Those who worked day and/or afternoon shifts, but no night shifts.

**Table S6.** Discrete-time proportional hazards model with estimated hazard ratio (HR) for first-time medically certified sickness absence due to CMDs for **nursing assistants only** in association with type of shift work and frequency of night shift among health care employees during the study period January 2009 - December 2020. We analysed the outcome as functions of working hour characteristics in the preceding year. The cut-off points for the distribution of exposure were based on quartiles in the distribution of the exposed group.

| Exposure | Person Years | No. of cases | Crude HR | Adjusted HR^a^ (95% CI) |
| --- | --- | --- | --- | --- |
| **Type of shift work (the full sample)** | | | | |
| Shift work, without night shift | 29367 | 719 | 1 | 1 |
| Shift work, with night shift | 13470 | 298 | 0.89(0.77-1.01) | 0.86(0.75-0.99) |
| Night work only | 3643 | 57 | 0.63(0.48-0.82) | 0.69(0.52-0.89) |
|  |  |  |  |  |
| **Frequency of night shifts (the full sample)** | | | | |
| Never night shifts^b^ | 29367 | 719 | 1 | 1 |
| 1-18 | 4324 | 97 | 0.90(0.72-1.10) | 0.84(0.68-1.04) |
| 19-49 | 4221 | 103 | 0.97(0.79-1.19) | 0.93(0.75-1.14) |
| 50-121 | 4498 | 81 | 0.73(0.58-0.92) | 0.74(0.59-0.93) |
| >121 | 4070 | 74 | 0.72(0.56-0.91) | 0.79(0.62-1.00) |
| Trend test |  |  |  |  |
| p-value trend |  |  |  |  |

^a^Adjusted for the time-dependent variables age and the fixed variables profession, sex, and country of birth. Quick return analyses were adjusted for night and afternoon shift counts, respectively. Adjustment for calendar year of follow-up is inherent in the analytical model.
^b^Those who worked day and/or afternoon shifts, but no night shifts.

**Table S7.** Linear estimation of the length of sickness absence (in terms of days) due to common mental disorders.

| Exposure | Unstandardised estimate | Standard error | t-value | p-value |
| --- | --- | --- | --- | --- |
| **Type of shift work** |  |  |  |  |
| Day work only | 0.00 | - | - | - |
| Shift work, without night shift | -33.15 | 13.76 | -2.41 | 0.01 |
| Shift work, with night shift | -28.75 | 15.16 | -1.90 | 0.05 |
| Night work only | -39.81 | 29.01 | -1.37 | 0.17 |
| **Frequency of night shifts** |  |  |  |  |
| Never night shifts | 0.00 | - | - | - |
| 1-18 | 13.41 | 12.32 | 1.09 | 0.27 |
| 19-49 | -18.26 | 14.45 | -1.26 | 0.20 |
| 50-121 | -46.86 | 15.73 | -2.98 | 0.00 |
| >121 | 15.51 | 21.02 | 0.74 | 0.46 |
| **Frequency of ≥3 consecutive night shifts** |  |  |  |  |
| 0 times | 0.00 | - | - | - |
| 1-4 | 8.66 | 19.68 | 0.44 | 0.65 |
| 5-12 | -5.51 | 21.88 | -0.25 | 0.80 |
| 13-21 | -29.30 | 24.40 | -1.20 | 0.23 |
| >21 | -0.34 | 27.47 | -0.01 | 0.98 |
| **Quick returns (<28 hours) from night shifts** |  |  |  |  |
| No quick returns from night shifts | 0.00 | - | - | - |
| 1-9 | -40.36 | 36.25 | -1.11 | 0.26 |
| 10-28 | -52.86 | 39.30 | -1.35 | 0.17 |
| 29-69 | -69.44 | 56.12 | -1.24 | 0.21 |
| >69 | -35.01 | 75.49 | -0.46 | 0.64 |
| **Quick returns (<11 hours) from afternoon shifts** |  |  |  |  |
| No quick returns from afternoon shifts | 0.00 | - | - | **-** |
| 1-17 | 28.90 | 21.60 | 1.34 | 0.18 |
| 18-32 | 33.43 | 21.03 | 1.59 | 0.11 |
| 33-49 | 28.51 | 21.50 | 1.33 | 0.18 |
| >49 | 15.72 | 23.89 | 0.66 | 0.51 |
